# DE-AFO: A Robotic Ankle Foot Orthosis for Children with Cerebral Palsy Powered by Dielectric Elastomer Artificial Muscle

**DOI:** 10.3390/s24123787

**Published:** 2024-06-11

**Authors:** Vahid Mohammadi, Mohammad Tajdani, Mobina Masaei, Sahel Mohammadi Ghalehney, Samuel C. K. Lee, Ahad Behboodi

**Affiliations:** 1 Department of Biomechanics, University of Nebraska Omaha, Omaha, NE 68106, USA; vmohammadi@unomaha.edu (V.M.); mmasaeikoroyeh@unomaha.edu (M.M.);; 2 Independent Researcher, Tehran 1417935840, Iran; mohammadtajdani@gmail.com; 3 Department of Physical Therapy, University of Delaware, Newark, DE 19716, USA

**Keywords:** exoskeleton, cerebral palsy, AFO, dielectric elastomer, gait, artificial muscle

## Abstract

Conventional passive ankle foot orthoses (AFOs) have not seen substantial advances or functional improvements for decades, failing to meet the demands of many stakeholders, especially the pediatric population with neurological disorders. Our objective is to develop the first comfortable and unobtrusive powered AFO for children with cerebral palsy (CP), the DE-AFO. CP is the most diagnosed neuromotor disorder in the pediatric population. The standard of care for ankle control dysfunction associated with CP, however, is an unmechanized, bulky, and uncomfortable L-shaped conventional AFO. These passive orthoses constrain the ankle’s motion and often cause muscle disuse atrophy, skin damage, and adverse neural adaptations. While powered orthoses could enhance natural ankle motion, their reliance on bulky, noisy, and rigid actuators like DC motors limits their acceptability. Our innovation, the DE-AFO, emerged from insights gathered during customer discovery interviews with 185 stakeholders within the AFO ecosystem as part of the NSF I-Corps program. The DE-AFO is a biomimetic robot that employs artificial muscles made from an electro-active polymer called dielectric elastomers (DEs) to assist ankle movements in the sagittal planes. It incorporates a gait phase detection controller to synchronize the artificial muscles with natural gait cycles, mimicking the function of natural ankle muscles. This device is the first of its kind to utilize lightweight, compact, soft, and silent artificial muscles that contract longitudinally, addressing traditional actuated AFOs’ limitations by enhancing the orthosis’s natural feel, comfort, and acceptability. In this paper, we outline our design approach and describe the three main components of the DE-AFO: the artificial muscle technology, the finite state machine (the gait phase detection system), and its mechanical structure. To verify the feasibility of our design, we theoretically calculated if DE-AFO can provide the necessary ankle moment assistance for children with CP—aligning with moments observed in typically developing children. To this end, we calculated the ankle moment deficit in a child with CP when compared with the normative moment of seven typically developing children. Our results demonstrated that the DE-AFO can provide meaningful ankle moment assistance, providing up to 69% and 100% of the required assistive force during the pre-swing phase and swing period of gait, respectively.

## 1. Introduction

Cerebral palsy (CP) is the most common pediatric neurological disorder, with the Centers for Disease Control and Prevention (CDC) estimating that about one in 345 children live with CP [1]. Ankle control deficiencies frequently manifest in CP and are the prominent contributors to gait abnormalities [2] that yield consequential outcomes, including an elevated risk of falls and injuries and inefficient gait speeds. The normal progression of CP is for overall mobility and walking function loss with aging. Walking ability directly impacts functional independence, physical activity, and social and community participation [3]. Additionally, impaired walking in this population puts individuals at risk for the development of preventable secondary health risks of inactivity and higher mortality rates. Adults with CP reported significantly greater prevalence rates of cardiovascular disease than those without CP [4,5].

Gait pathologies typically worsen in CP over time, with many children becoming more dependent on assistive devices as they age into adulthood [6,7,8,9]. However, the standard of care for ankle control dysfunction associated with CP is an unmechanized, bulky, and uncomfortable L-shaped passive ankle foot orthosis (AFO). The AFO designs have not undergone substantial advances in decades and are primitive compared to advancements in prosthetics technology. Although successful in providing passive ankle stability, long-term use of these orthoses often constrains the ankle’s motion and, consequently, causes disuse atrophy of muscles, skin damage, and adverse neural adaptations [10,11]. Ring et al. [12] noted that AFOs block normal ankle kinematics and prevent active ankle stability during gait. The inhibition of sensory feedback needed for motor control is another side-effect of conventional AFO use [13].

Alternatively, powered orthoses hold the potential for enhanced independence and the re-education of the neuro-motor system, consequently reducing individual reliance on orthotic devices [14]. Several powered AFOs have been proposed, with substantial torque generation capabilities to assist the plantarflexor muscles in pushing the center of mass forward [15,16,17,18]. These AFOs are predominantly powered by pneumatic actuators [19,20,21] or direct current (DC) motors [22,23], which are not evolved for working in the vicinity of the human body and, therefore, incorporate inflexible mechanical frameworks that impede natural joint movement and often result in pressure sores on users’ limbs. Because their actuators are not evolved for working in the vicinity of the human body, their rigidity, bulkiness, heaviness, and acoustically noisy design diminishes user acceptability and comfort, which inhibits their commercialization and adaptation. Thus, employing actuators specifically designed to mimic biological muscles, i.e., artificial muscles, is receiving greater attention.

Cable-driven exoskeletons powered by DC motors have emerged as more compact and more flexible solutions. Commercially available devices like ReWalk Restore [24] and Biomotume [25] demonstrate this trend. However, the assistance provided by these devices is limited to the sagittal plane, which is insufficient in addressing the complex gait deviations common to individuals with CP. Consequently, the Biomotume is primarily utilized for gait training [26,27]. In a novel approach, Park et al. introduced a soft-powered AFO that closely mimicked the musculoskeletal structure of the ankle joint [19]. Their bio-inspired orthosis facilitated motion in both the sagittal (plantarflexion/dorsiflexion) and frontal planes (eversion/inversion). However, such proposed pneumatic AFOs are heavy, approximately 2.5 kg in total weight, and generate acoustic noise of about 65 dB [28,29], with the annoyance threshold at 70 dB (e.g., that of a washing machine) [30], as indicated by the CDC. Recently, more portable variants of soft ankle foot orthoses (AFOs) have been developed that not only assist the ankle in the sagittal plane but also provide active mediolateral support, such as eversion and inversion resistance and assistance, utilizing cable-driven [31,32] or pneumatic [33] designs. While still somewhat conspicuous, these designs show promise as viable powered AFO alternatives in the near future. Finding a soft, lightweight, noiseless, and compact alternative muscle-like actuator is paramount for rehabilitation robotics.

Through participating in the National Science Foundation (NSF) I-Corps (project #1906128), we successfully interviewed 185 stakeholders and identified essential features for designing a user-compliant AFO [34]. For AFO users, comfort is the main issue in conventional designs. The conspicuousness of the conventional AFOs was mentioned by 48% of customers, mostly parents. This issue is exacerbated as the user enters the teenage years, mainly because it attracts attention, decreases usage time, and eventually leads to abandonment. The device’s bulk substantially limits subjects’ shoe choices and usually binds them to use “ugly” sneakers. The rigidity, which creates pressure points and heat accumulation due to the non-breathable material, causes extreme discomfort and skin damage; 47% of customers noted these issues. The resulting discomfort makes the walking experience uncomfortable and consequently decreases the device’s usage time. Furthermore, 38% of physical therapists express the need for a smart device that can substantially assist the subject in pushing off the ground during walking, which can improve walking efficiency, endurance, and speed. A subject-specific device that can readily fit and assist different types of foot deformities and gait deviations was also crucial for 29% of the customers, mostly orthotics and physical therapists, to reduce the number of fitting/adjustment sessions. This analysis drove our design criteria.

**Figure 1:**
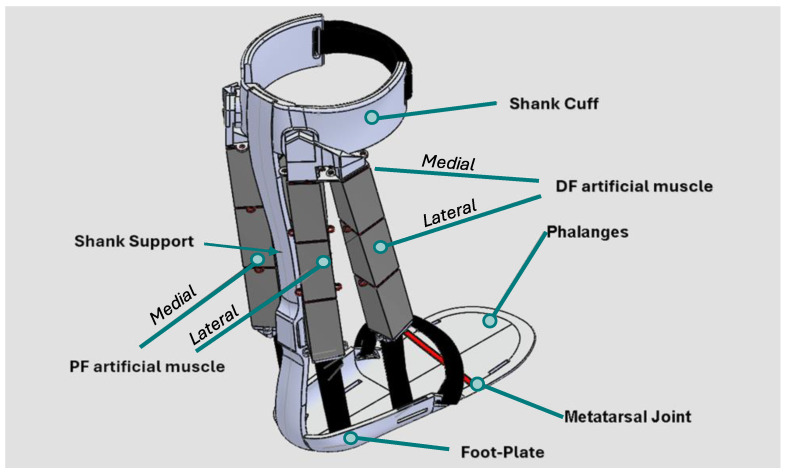
DE-AFO and its building blocks include medial and lateral plantarflexor (PF) artificial muscle, metatarsal joint, and medial and lateral dorsiflexor (DF) artificial muscle.

Because previously designed AFOs for children with CP are often passive and uncomfortable and do not meet the needs and wants of many stakeholders, our objective in this study was to design a comfortable, smart AFO for children with CP powered by a soft and comfortable actuator called dielectric (DE). DE actuators are a group of muscle-like actuators (artificial muscles) that are lightweight, compact, soft, noiseless, and contract longitudinally. We call this device DE-AFO [35] (Figure 1), a biomimetic exoskeleton that synchronizes the activation of its artificial muscles with those of the ankle by detecting different phases in gait to promote natural ankle motion. Our main hypothesis was that DEA-based artificial muscles in DE-AFO can generate meaningful assistive force in the sagittal plane to improve ankle movements in children with CP. Therefore, the main outcome measure was dorsiflexion and plantarflexion assistive force generated in DE-AFO compared with the force required to produce a typical ankle moment. In feasibility analysis, we aimed to explain the design of DE-AFO and theoretically calculate its force generation capabilities in a case study as a first step toward proving our hypothesis. To this end, (1) we will explain the artificial muscle that powers DE-AFO; (2) we will present the mechanical structure of the DE-AFO that holds these muscles around the ankle; and (3) we will demonstrate the force profile of DE-AFO using the ankle dynamic of a representative child with CP.

### 1.1. Design Criteria

The successful design of a pediatric ankle exoskeleton hinges on a thorough understanding of the human ankle’s anatomical, biomechanical, and developmental specifics in children. Through these considerations and incorporating the voices of stakeholders, we can create an effective AFO with limited to no adverse effects on the rehabilitation process and improve the quality of life for young users.

The sensory feedback from the ankle is critical for developing motor skills and balance. The design of a pediatric ankle exoskeleton should not hinder this development, aiding in the proprioceptive and sensory-motor development crucial for walking. Only a comfortable, smart design that can synchronize its motion to a child’s gait and mimic the natural sensory inputs of the ankle can limit the adverse effects and contribute to the rehabilitation and motor development processes. Above all features, we aim to design a powered AFO that is comfortable and unobtrusive. Additionally, through our customer discovery, we identified five innovative features in designing DE-AFO, which include the following:

1. **Low-profile Foot Segment**: Enhances the design’s comfort and conspicuousness by allowing standard shoe sizes and styles, unlike traditional AFOs that often require oversized shoes;
2. **Modularity and Scalability:** Children’s bones, ligaments, and tendons are still developing, which influences both the flexibility and the resilience of the ankle joint. The design of an ankle exoskeleton for children must accommodate this growth, ensuring that the device is adjustable and does not impede natural development. Moreover, the variability in growth rates among children necessitates a modular approach to exoskeleton design, allowing for customization and scalability. In addition, it needs to be adaptable to individual needs due to the variability in CP symptoms and rapid pace of growth in the target age range, 3 to 10 years old (young kids) who are typically under 30 kg in weight per CDC growth charts. The modular design enables customization to the user’s specific requirements and allows for easy replacement of parts due to wear and tear, growth, or changes in functional ability. This reduces the need for frequent replacements and associated costs;
3. **Providing Meaningful Assistance to Ankle Muscles:** An effective AFO must provide the necessary torque for a child with CP to facilitate walking. Therefore, the Results Section will focus on theoretically evaluating if DE-AFO can deliver the required deficient ankle in a representative child with CP during the pre-swing and swing phases of gait, ensuring effective propulsion and safer movement;
4. **Smart Adaptability to Users’ Gait**: The DE-AFO must feature real-time responsiveness during walking to synchronize itself with the ankle dynamic, thereby assisting gait, enhancing mobility and comfort, and promoting sensorimotor development.
5. **Metatarsal Joint:** Current AFO designs do not incorporate metatarsal articulation. An improved design should include this feature at the footplates to provide a more natural push-off during gait.
6. **Active Mediolateral Support**: Conventional AFOs often use uncomfortable rigid structures to create mediolateral stability. Current commercial low-profile AFOs, like the Noodle AFO [36] by Kinetic Research, offer sleek designs but fail to provide adequate mediolateral support. The support is critical for more impaired users and may increase preferred walking speeds [37], and lower the physiological cost of walking [38].

By integrating these considerations, we aim to enhance the acceptability of DE-AFO, promote natural ankle motion, and, thereby, allow for proper sensory and motor development.

### 1.2. Ankle Dynamic

The ankle has three major roles in walking: (1) creating stability during the stance period of gait; (2) forward progression, primarily during the push-off phase of gait through activation of the plantarflexor muscles; and (3) clearing the foot off the ground during the advancement of the leg forward (swing phase) by dorsiflexor muscles.

**Figure 2:**
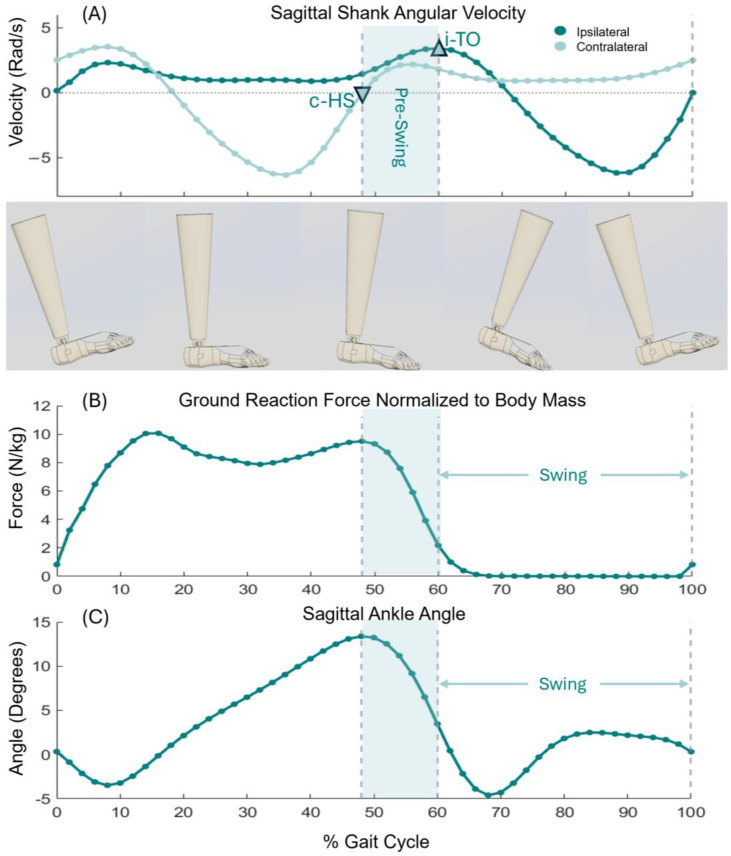
Ankle dynamic. Sagittal plane shank angular velocity (**A**), ground reaction force (**B**), and ankle angle (**C**) for typically developing kids (TDs) during a complete gait cycle, divided into four phases. The horizontal axis represents the percentage of the gait cycle, ranging from 0% to 100%. The downward triangle (**A**) represents the event (zero crossing from negative to positive on the contralateral side) associated with the contralateral heel strike (c-HS) and the initiation of ipsilateral pre-swing. The upward arrow identifies the event (the first prominent peak after the onset of pre-swing) associated with ipsilateral toe-off (i-TO).

In the design of an ankle exoskeleton, understanding the dynamics of the ankle motion, especially the ankle’s angular velocity (Figure 2A), moment, and ground reaction force (GRF) during walking, is pivotal for promoting natural gait patterns and ensuring the device’s effectiveness in enhancing mobility. The ankle joint plays a crucial role in locomotion as a pivotal point that manages GRF, supports body weight, and facilitates forward movement.

The GRF at the ankle (Figure 2B) is a complex interplay of biomechanical forces that vary throughout the gait cycle. Initially, during the heel strike phase, the ankle experiences a rapid increase in GRF as the foot contacts the ground, absorbing the impact and supporting the body’s weight. This phase is characterized by a relatively low ankle GRF, which gradually increases as the foot transitions to flat on the ground. As the gait cycle progresses to the midstance phase, the body’s center of mass moves over the foot, and the GRF peaks. Generation of this peak force is crucial for the push-off phase, during pre-swing, where the stored elastic energy in the Achilles tendon and the contraction of plantarflexor muscles propels the body forward. Thus, the exoskeleton must assist with its full capacity during pre-swing to facilitate natural and efficient walking. In the final phases of the gait, the ipsilateral toe starts to leave the ground, and GRF decreases rapidly as initiating the swing period at toe-off, clearing the foot off the ground, and preventing the patient from tripping and falling using the dorsiflexor muscle. Here, the exoskeleton’s design must ensure minimal resistance and flexibility to allow for a smooth transition to the next step.

Accurately replicating the ankle moment generated by the GRF during the design of an ankle exoskeleton is crucial for promoting natural ankle functions. This involves using advanced actuators and control strategies to adapt to the user’s walking patterns, providing support and assistance without hindering natural movement.

## 2. Methods

Our approach had three major components: (1) identifying and employing an alternative muscle-like actuator (artificial muscle); (2) developing a control system that can synchronize the activation of the artificial muscle to natural ankle motion; and (3) an appropriate mechanical structure.

After a thorough investigation of candidate soft actuators for pediatric rehabilitation robotics, we selected stacked dielectric elastomer actuators (DEAs) for configuring our artificial muscle, which are detailed in the following subsection [39]. Artificial muscle was the primary component that met our design criteria. Our DE-AFO design, shown in Figure 1, incorporates artificial muscles along the shank support bar located posterior to the ankle to assist the plantarflexor muscles. This artificial muscle consists of two artificial myofibrils in parallel, located medially and laterally, each containing three stacked DEAs in series; this muscle plays a key role in generating the required assistive force during pre-swing to facilitate plantarflexion of the foot for effective push-off. Additionally, we have utilized another artificial muscle located anterior to the ankle to assist the dorsiflexor muscles. This dorsiflexor artificial muscle has two myofibrils located laterally and medially. Like the plantarflexor artificial muscle, each myofibril consists of three stacked DEAs. At the onset, DE-AFO’s artificial muscles are contained in flexible brackets, printed using thermoplastic polyurethane (TPU) filaments and flexible, non-expandable bands to fasten the artificial muscle to the DE-AFO’s footplate securely. The entire mechanism weighs 400 g.

### 2.1. DE-Based Artificial Muscle (Smart, Comfortable, Lightweight, Acoustically Noiseless, Compact with Linear Contraction)

**Figure 3:**
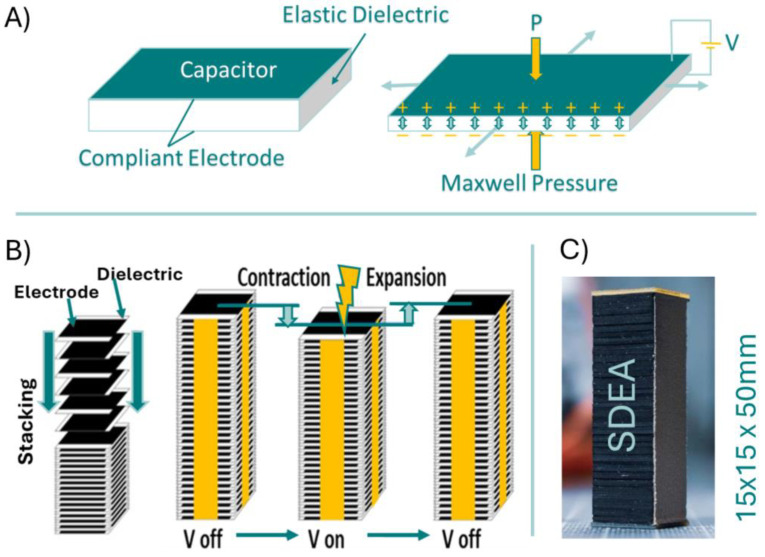
(**A**) Elastic capacitor and Maxwell pressure creating displacement. (**B**) Stacked dielectric elastomer actuator (SDEA) configuration consists of multiple layers of elastic capacitors. (**C**) A real SDEA consists of about 2500 layers of capacitors.

Soft electroactive polymer actuators, such as DEAs, offer rapid, efficient, and lightweight alternatives to conventional DC motor actuators in rehabilitation robotics, operating quietly as well. DEAs, which closely mimic the behavior of natural muscles, are highly valued and extensively studied within the field of soft actuators [40,41,42]. These actuators are composed of a flexible dielectric material placed between two compliant electrodes, forming a stretchable capacitor (Figure 3A). Application of a DC voltage across the electrodes generates electrostatic forces, known as Maxwell pressure, which compress the dielectric and induce contraction of the actuator (Figure 3A). DEAs are distinguished by their exceptional power-to-mass ratios, surpassing those of human skeletal muscles, and feature rapid response times, inherent length self-sensing, and energy recuperation capabilities [43]. Furthermore, they demonstrate high efficiency, with low energy consumption primarily due to minimal dielectric leakage currents (in less than 10 mA range) when the capacitor is fully charged [44]. Mechanically, a DE actuator functions as a variable-stiffness spring, while electrically, it is a capacitor [45].

Allen et al. have designed a remarkable DE-powered AFO that achieved an impressive 49% dorsiflexion during gait [45]. However, the design did not provide ample ankle stability required for children with CP and did not have plantarflexion assistance. The design relied on surface area expansion rather than longitudinal contraction, which might be a limiting factor in power generation efficiency.

#### Introducing Stacked DEAs and the Benchmark Tests

By stacking multiple layers of enhanced DEA, Kovacs et al. [46] devised a highly adaptable actuator resembling human skeletal muscle. Being lightweight, compact, soft, and noiseless, and contracting longitudinally, stacked DEAs are strong artificial muscle candidates [39]. Specifically, stacked DEAs, composed of multiple layers of elastomer coated with compliant electrodes, contract longitudinally when voltage is applied (Figure 3B). A stacked actuator with a 39 mm height and 17 × 17 mm planes with an elastic modulus of 1.4 MPa, under 1200 V driving voltage, demonstrated a maximum force of 10 N (almost 32 kPa) and a 5% unidirectional strain [44]. Here, we propose an AFO design based on stacked DEAs [44] that may improve user compliance. As discussed above, the artificial muscles of DE-AFO consist of stacked DEAs as their motor units.

As the first step of the design, we benchmark-tested the mechanical properties of stacked DEAs, including the force and displacement capability of the commercially available stacked DEAs [44] and the configured artificial muscles. Each can produce 10 N of force and close to 4% of shortening in their 2016 version. An artificial muscle is formed by configurations of stacked DEAs, in series and parallel, depending on the needed length of shortening and actuation force, respectively. The 1 × 3 artificial muscle, i.e., an artificial muscle of one myofibril, consisting of three stacked DEAs in series, like the lateral myofibril of the dorsiflexor artificial muscle (Figure 1), could generate 10.18 N of force and 3.53 mm of shortening. Note that in the 2023 version, the shortening is about 8% while force generation increased to 12 N. A stacked DEA-based artificial muscle improves the comfort and inconspicuousness of the device and fulfills our design criteria:

1. **Low-profile Foot Segment**: The artificial muscles contract longitudinally and will be connected via a flexible, non-expandable band to the footplate, reducing the bulk around the foot and allowing the user to wear a normal size shoe;
2. **Modularity and Scalability:** By employing stacked DEAs, we configured a modular artificial muscle. The number of stacked DEAs, i.e., motor units, and their configurations in the artificial muscle can be adjusted to custom fit the force and displacement required for accommodating child growth and gait abnormalities. By increasing the number of stacked DEAs in each myofibril, i.e., stacked DEAs connected in series, we can increase the artificial muscles’ displacement. By adding myofibrils in parallel, we can create stronger muscle fibers.

The third criterion, **Providing Meaningful Assistance to Ankle Muscles**, will be evaluated in the Results Section.

### 2.2. Control System: Fourth Criterion of Smart Adaptability to Users’ Gait

The major component of the DE-AFO is its control system, which features real-time responsiveness to ankle motion during walking. Equipped with an inertial measurement unit (IMU), which streams shank angular velocity (Figure 2A) to DE-AFO, we have developed a finite state machine that detects the seven major phases of gait using this input. The phases include the stance phases: loading response, mid-stance, terminal stance, and pre-swing, and the swing phases: initial swing, mid-swing, and terminal swing. This enables the artificial muscles to be in sync with the ankle’s plantarflexion and dorsiflexion contraction. Our gait phase detection algorithm can detect all seven gait phases. We evaluated this state machine on typically developing children (TD) and healthy adults [47]. This algorithm was also tested in real time on seven typically developing children and six children with CP, showing 99% detection reliability [48,49].

In designing the DE-AFO, our focus is on providing plantarflexion assistance during the pre-swing to improve propulsion and dorsiflexion assistance during the swing phases to prevent trips and falls. Pre-swing initiates when the contralateral heel hits the ground (heel strike); the state machine detects the heel strike when the contralateral shank angular velocity changes direction from negative (counter-clockwise) to positive (clockwise) (downward triangle in Figure 2A). The swing period starts with the initial swing phase of gait when the ipsilateral toe leaves the ground (toe-off). The state machine detected toe-off using the first prominent peak after the contralateral heel strike (upward triangle in Figure 2A).

### 2.3. Mechanical Structure

For the second criterion, modularity and scalability, we designed a mechanical structure with three main components: the cuff, shank support, and footplate. Each component can be individually scaled and adjusted in concert with the artificial muscles. Furthermore, the muscles are attached to the cuff using a magnetic snap-in mechanism, which facilitates easy attachment and detachment of the muscles. This design feature simplifies the process of wearing the device and adjusting the muscles’ force and displacement by increasing the number of motor units and stacked DEAs.

To address the fifth criterion, the metatarsal joint. The current DE-AFO utilizes a soft hinge joint, printed from TPU filaments, that connects the back of the footplate to its phalange section (Figure 1). This hinge allows for metatarsal articulation, providing a more natural push-off during gait, and thereby enhances the overall walking efficiency.

### 2.4. Evaluation of DE-AFO Assistance in Sagittal Plane

To evaluate the DE-AFO’s capability in assisting ankle moments, we calculated the difference between the ankle moment of a subject with CP and that of TDs. We then measured the required plantarflexion and dorsiflexion assistive forces to overcome this deficit. To validate whether the DE-AFO can provide meaningful assistance in the sagittal plane, we compared these forces to the plantarflexion force generated by the DE-AFO during pre-swing and the dorsiflexion force during swing. We defined meaningful assistance as the ability to provide at least 50% of the required force to overcome the deficit.

The utilized CP and TD data were previously collected in two different studies. The normative ankle moment of TD data is an average of seven participants (12 ± 1 years of age, four males). The representative CP subject is an 11-year-and-5-month-old boy weighing 25.89 kg. All subjects signed informed consent forms. Study procedures were approved by the University of Delaware Institutional Review Board. Consent and assent were obtained from participants. Table 1 summarizes the information on our study samples.

The participants walked on a treadmill while reflective markers were attached to their lower limbs’ anatomical landmarks. The gait dynamic was recorded using instrumented motion capture (Motion Analysis Corporation, Santa Rosa, CA, USA) with a sampling rate of 128 Hz and two force places (Bertec, Columbus, OH, USA) with a sampling rate of 3200 Hz. The data were processed in Visual 3D (C-Motion Inc., Germantown, MD, USA).

## 3. Results

### Evaluating the Third Criterion

**Figure 4:**
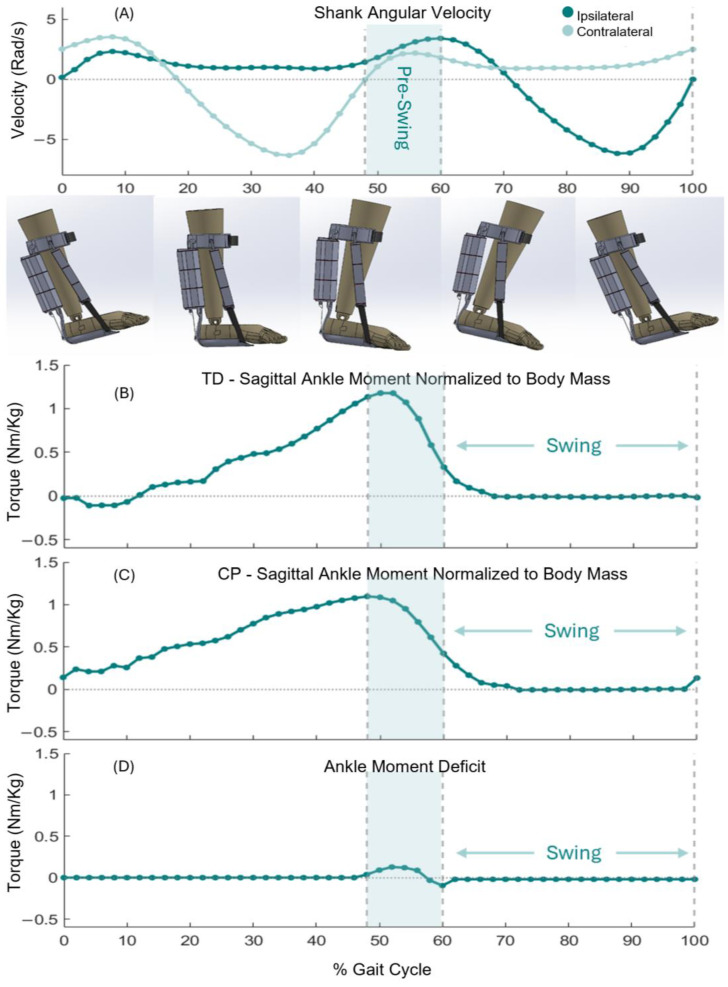
Ankle dynamic. (**A**) Sagittal plane shank angular velocity, (**B**) Typically developing kids’ (TDs’) normative ankle moment in sagittal plane, an average of seven TDs. (**C**) That of a representative child with cerebral palsy (CP, C). (**D**) The deficit moment is calculated by subtracting TD from the CP moments.

Here, the objective is to calculate the deficit (Figure 4D) in the ankle moment of the CP subject (Figure 4C) and that of TDs (Figure 4B) and measure the force that each plantarflexor and dorsiflexor artificial muscle needs to generate, based on a real-world scenario. Thereby, we evaluated the capability of the design to assist ankles in children with CP.

(1) $$\tau_{TD}=\tau_{Exo}+\tau_{CP}$$

(2) M=24 kg H=137 cm

**Figure 5:**
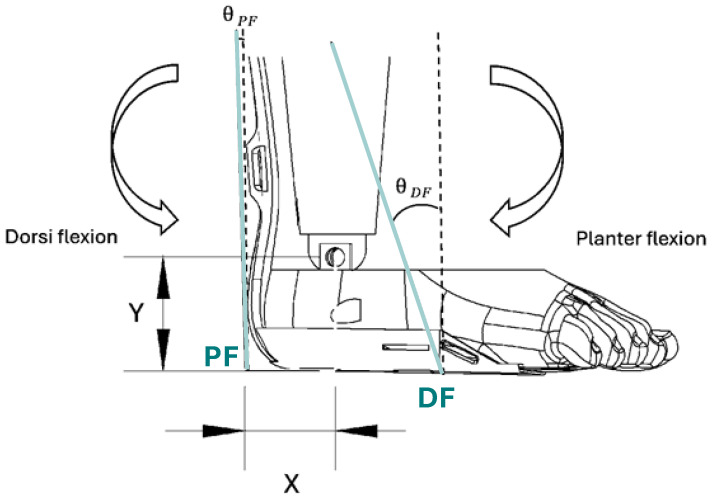
Ankle location (X, Y) relative to the DE-AFO. Clockwise rotation of the ankle is called plantarflexion. DF and PF indicate the insertion point of the artificial muscle. Θ indicates the artificial muscle angle to the vertical axis.

Then, using anthropometric data and the subject’s height, H = 130, we calculated the length of the foot, L [50], ankle height, Y [51], and heel-to-ankle distance, i.e., PF artificial muscle lever arm, X [51] (Figure 5).

(3) Y=0.39×H=44 mm

(4) L=0.152×H=208

(5) X=0.018×L=38 mm

The location where forces are applied on the footplate for the plantarflexor (PF) and dorsiflexor (DF) artificial muscles are as follows:

(6) $$L_{DF}=L_{Shank}\times \tan\left(\theta_{DF}\right)$$

(7) $$L_{PF}=L_{Shank}\times \tan\left(\theta_{PF}\right)$$

where θ is defined as the angle between the artificial muscles and superior–inferior access. Using the ankle angle, $\theta_{Ankle}$ (Figure 2C), we calculated the length of the front actuators, $L_{DF}$, and length of the rear actuators, $L_{PF}$, using the following equations:

(8) $$L_{DF}=\sqrt{L_{Shank}^{2}+L_{DF}^{2}-2\times L_{Shank}\times L_{DF}\times cos(\theta_{Ankle})}$$

(9) $$L_{PF}=\sqrt{L_{Shank}^{2}+L_{PF}^{2}-2\times L_{Shank}\times L_{PF}\times cos(\theta_{Ankle})}$$

The following equations represent the angles at which forces are applied during walking relative to the footplate:

(10) $$\sin\left(\theta_{PF}\right)=\frac{L_{Shank}\times \sin\left(\theta_{Ankle}\right)}{L_{PF}}$$

(11) $$\sin\left(\theta_{DF}\right)=\frac{L_{Shank}\times \sin\left(\theta_{Ankle}\right)}{L_{DF}}$$

The desired forces are the following:

(12) $$F_{PF}=\frac{\tau_{EXO}}{\sin\left(\theta_{PF}\right)\times L_{PF}}$$

(13) $$F_{DF}=\frac{\tau_{EXO}}{\sin\left(\theta_{DF}\right)\times L_{DF}}$$

**Figure 6:**
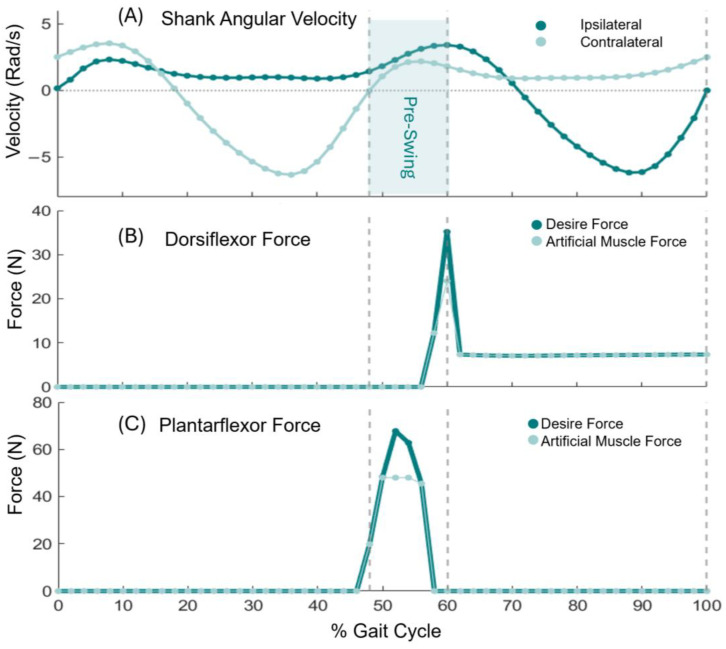
(**A**) Ankle dynamic. Sagittal plane shank angular velocity, Desire force vs Dorsiflexion Force (max 24 N) (**B**) and Plantarflexion force (max 48 N) (**C**) are generated by the artificial muscles of DE-AFO.

**Figure 7:**
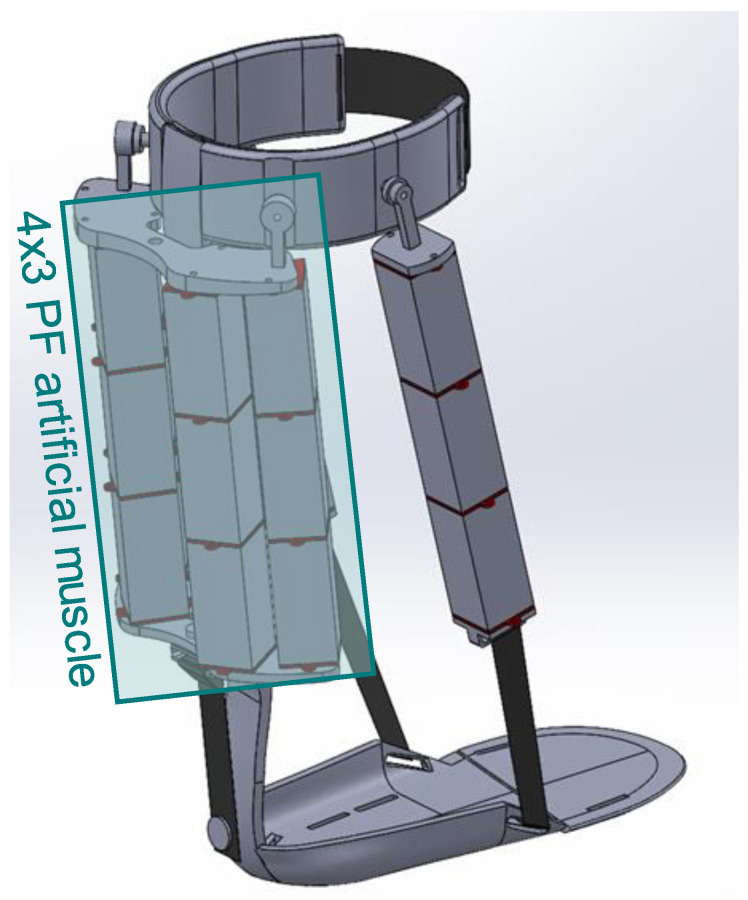
A DE-AFO with 4 × 3 plantarflexor (PF) artificial muscle capable of generating 48 N plantarflexion force.

The maximum required force for assisting plantarflexion during pre-swing was calculated at 70 N, F_PF_. Leveraging the modularity of the DE-AFO, we custom-fit the plantarflexor artificial muscles to produce a substantial portion of the required force. To prevent excessive bulk around the shank, we configured a muscle capable of producing 48 N of force, i.e., ~70% of the required force (Figure 6B). The muscle was configured by assembling four stacked DEA myofibrils in parallel, each consisting of three stacked DEAs in series (4 × 3 artificial muscle, Figure 7). We thereby fulfill our third criterion: providing meaningful assistance to ankle muscles.

## 4. Conclusions

In this study, we introduced the DE-AFO, the first AFO powered by DE-based artificial muscles. Through our customer discovery process, we identified a need for a more comfortable and discreet design, leading to the formulation of six key design criteria: (1) low-profile foot segment; (2) modularity and scalability; (3) meaningful assistance to ankle muscles; (4) smart adaptability to users’ gait; (5) inclusion of a metatarsal joint; and (6) active mediolateral support. We addressed most of these criteria effectively using DE-based artificial muscles and a gait phase detection system tailored for children with cerebral palsy (CP). The acoustically noiseless operation of the artificial muscle and their longitudinal contraction enhanced the consciousness of the current-powered AFOs. The light weight and softness of the stacked DEAs improved the comfort. Our design incorporates modular artificial muscles, structured similarly to skeletal muscle, which includes motor units and myofibrils to fine-tune shortening and force generation. However, due to concerns about the conspicuousness of the design of the device, there are limitations on the number of motor units, stacked DEAs, and modules that can be configured in series and in parallel.

Our primary finding involved comparing the force profile of the DE-AFO around the ankle in the sagittal plane with the assistive force required to generate a typical ankle moment. The DE-AFO successfully compensated for 69% of the plantarflexion force deficit during the pre-swing phase and 100% of the required force throughout most of the swing phase. This enabled a child with CP to achieve near-typical ankle motion. Despite the limitation of reporting on only one case of CP in this study, this work represents a pivotal step in validating our hypothesis that the DE-AFO can provide meaningful assistive force in the sagittal plane to achieve typical ankle moments. Our findings grant further investigation and the progression towards conducting a fully powered clinical trial.

Before initiating clinical trials, the next milestone will involve rigorous benchmark testing of the DE-AFO on an ankle model. As recommended by the FDA in our pre-sub meeting, we will first test the DE-AFO using an instrumented phantom ankle model we developed. This preliminary testing will ensure the safety and efficacy of the device before it is tested on children. This ankle model incorporates load cells and torque sensors at strategic points such as the medial and lateral malleoli and under the heel. This testing will measure the moment generated by the artificial muscles on the ankle, providing preliminary data before clinical trials on typically developing children and those with CP. We are modifying and instrumenting our previously designed bipedal robot [52] to don the DE-AFO and serve as a test rig for analyzing the DE-AFO’s dynamic behavior during walking in parallel with benchmark testing.

A significant future focus will be enhancing the active mediolateral support—our fourth design criterion. Current commercial low-profile AFOs, like the Noodle AFO by Kinetic Research, offer sleek designs but fail to provide adequate mediolateral support, which is critical for more impaired users. By adjusting the stiffness of the artificial muscles isometrically around the ankle, the DE-AFO aims to provide enhanced support during stance and reduce pressure on the ankle during the swing phase of gait to improve comfort, a feature not adequately addressed by rigid AFOs. We plan to develop the first active and comfortable mediolateral support system that could potentially increase preferred walking speeds and decrease the energetic cost of walking.

In addition to its impact on children with CP, incorporating active mediolateral support into the DE-AFO could significantly benefit other populations with ankle control deficits, such as individuals with chronic ankle instability (CAI). CAI is characterized by reduced control of ankle eversion and inversion during single-leg stance [53,54]. The DE-AFO’s advanced capability to detect stance phases allows it to precisely control the activation of its medial and lateral myofibrils, thereby stabilizing the ankle during the mid-stance and terminal stance phases of gait. This targeted support will enhance stability and reduce the risk of further injury for individuals with CAI.

We will also develop a hybrid DE-AFO that integrates a low-profile DC motor with the DE-AFO system, which will further enhance assistance and impedance control by precisely measuring torque, monitoring the current and angular displacement around the ankle [55]. Additionally, we can leverage the self-sensing capability of the stacked DEAs as well for tracking ankle trajectory [56,57].

## Figures and Tables

**Table 1:** Subjects include age, gender, self-selected walking speed (SSWS), height, and weight.

	Age (years)	Gender	SSWS (m/s)	Height (m)	Weight (kg)
TD01	16	M	0.8	1.78	71.92
TD02	10	M	0.8	1.46	32.55
TD03	10	F	1.2	1.46	31.95
TD04	12	F	1.25	1.59	43.25
TD05	12	F	1	1.47	36.42
TD06	14	F	1.1	1.55	52.61
TD07	13	F	1.1	1.73	56.29
CP01	12	M	0.9	1.37	25.89

## Data Availability

The raw data supporting the conclusions of this article will be made available by the authors on request.

## Abbreviations

CP	Cerebral Palsy
AFO	Ankle Foot Orthosis
DE	Dielectric Elastomer
DEA	DE Actuator
TD	Typically Developing
GRF	Ground Reaction Force
